# Low birth weight: Case definition & guidelines for data collection, analysis, and presentation of maternal immunization safety data

**DOI:** 10.1016/j.vaccine.2017.01.049

**Published:** 2017-12-04

**Authors:** Clare L. Cutland, Eve M. Lackritz, Tamala Mallett-Moore, Azucena Bardají, Ravichandran Chandrasekaran, Chandrakant Lahariya, Muhammed Imran Nisar, Milagritos D. Tapia, Jayani Pathirana, Sonali Kochhar, Flor M. Muñoz, The Brighton Collaboration Low Birth Weight Working Group

**Affiliations:** a Medical Research Council: Respiratory and Meningeal Pathogens Research Unit, Johannesburg, South Africa; b Department of Science and Technology National Research Foundation, Vaccine Preventable Diseases, South Africa; c Faculty of Health Sciences, University of the Witwatersrand, Johannesburg, South Africa; d Global Alliance to Prevent Prematurity and Stillbirth (GAPPS), Seattle Children’s Research Institute, Seattle, WA, USA; e Sanofi Pasteur Inc., Swiftwater, PA, USA; f ISGlobal, Barcelona Ctr. Int. Health Res. (CRESIB), Hospital Clínic – University of Barcelona, Barcelona, Spain; g Madras Medical College, India; h Department of Community Medicine, GR Medical College and Associated Hospitals, Gwalior, MP, India; i Department of Pediatrics and Child Health, Aga Khan University, Karachi, Pakistan; j University of Maryland School of Medicine, Center for Vaccine Development, MD, USA; k Global Healthcare Consulting, India; l Baylor College of Medicine, Departments of Pediatrics, Molecular Virology and Microbiology, Houston, TX, USA; m Erasmus University Medical Center, Rotterdam, The Netherlands

**Keywords:** Low birth weight, Adverse event, Immunization, Guidelines, Case definition

## 1 Preamble

### 1.1 Need for developing case definitions and guidelines for data collection, analysis, and presentation for low birth weight as an adverse event following maternal immunization

The birth weight of an infant is the first weight recorded after birth, ideally measured within the first hours after birth, before significant postnatal weight loss has occurred. Low birth weight (LBW) is defined as a birth weight of less than 2500 g (up to and including 2499 g), as per the World Health Organization (WHO) [1]. This definition of LBW has been in existence for many decades. In 1976, the 29th World Health Assembly agreed on the currently used definition. Prior to this, the definition of LBW was ‘2500 g or less’. Low birth weight is further categorized into very low birth weight (VLBW, <1500 g) and extremely low birth weight (ELBW, <1000 g) [1]. Low birth weight is a result of preterm birth (PTB, short gestation <37 completed weeks), intrauterine growth restriction (IUGR, also known as fetal growth restriction), or both.

The term low birth weight refers to an absolute weight of <2500 g regardless of gestational age. Small for gestational age (SGA) refers to newborns whose birth weight is less than the 10th percentile for gestational age. This report will focus specifically on birth weight <2500 g. Further details related to case definitions for PTB [2], IUGR and SGA are included in separate GAIA reports.

Globally, it is estimated that 15–20% of all births, or >20 million newborns annually, are low birth weight infants. Low- and middle-income countries account for a disproportionate burden of LBW; over 95% of the world’s LBW infants are born in LMICs. There are marked global and regional variations in LBW rates. An estimated 6% of infants are born LBW in East Asia and the Pacific, 13% in Sub-Saharan Africa, and up to 28% in South Asia [3]. Up to half of all LBW infants are born in south Asia [4]. High-income regions report lower LBW rates, including 6.9% from UK [5]. Of concern is the estimated increase in LBW rates in certain middle-income countries such as Oman, where the LBW rate went from 4% in 1980 to 8.1% in 2000 [6].

One of the major challenges in monitoring the incidence of LBW is that more than half of infants in the LMICs are not weighed [7]. Population-based survey data often rely on modeled estimates, with statistical methods to adjust for underreporting and misreporting of birth weight. In the context of vaccine safety monitoring, accurate ascertainment of birth weight in LMICs will continue to require attention and investment to improve accuracy and reporting of this important health indicator.

#### 1.1.1 Why are we concerned about low birth weight?

Low birth weight is a valuable public health indicator of maternal health, nutrition, healthcare delivery, and poverty. Neonates with low birth weight have a >20 times greater risk of dying than neonates with birth weight of >2500 g [8], [9]. Additionally, low birth weight is associated with long-term neurologic disability, impaired language development [10], impaired academic achievement, and increased risk of chronic diseases including cardiovascular disease and diabetes. Preterm infants carry additional risk due to immaturity of multiple organ systems, including intracranial hemorrhage, respiratory distress, sepsis, blindness, and gastrointestinal disorders. Preterm birth is the leading cause of all under-5 child mortality worldwide [11].

In addition, economic studies in low-income settings have demonstrated that reducing the burden of low birth weight would have important cost savings both to the health system and to households [12].

#### 1.1.2 What leads to low birth weight?

The underlying causes of both PTB and IUGR are multifactorial, and the biological pathways and preventive strategies for these two conditions are quite different [13], [14], [15]. The exact cause of PTB may be unknown in many cases, however numerous maternal, fetal and placental factors may contribute to PTB [13]. Significant maternal conditions include extra-uterine infection, chorioamnionitis, trauma and illness (e.g. pre-eclampsia/eclampsia). Significant fetal conditions include IUGR, fetal infection, death and anomalies. Placental pathologic conditions include placental abruption and placenta praevia [13].

In general, the causes of IUGR can be due to maternal, fetal, and placental factors. Although the etiologies are different, they often have the final common pathway of insufficient uterine-placental perfusion and fetal nutrition.

IUGR can be asymmetrical IUGR (where babies have features of malnutrition), symmetrical IUGR (hypoplastic small for dates) or mixed IUGR. Asymmetrical IUGR is the most common (70–80%) form of IUGR, resulting from an insult (often utero-placental insufficiency) later in pregnancy, which results in affected babies having normal length and head circumference (brain sparing), but reduced weight. Symmetrical IUGR on the other hand arises from an insult (often genetic, structural or infectious) occurring earlier in pregnancy leading to a reduction in all anthropometric parameters in fetus/newborn [15].

Insufficient perfusion, through abnormal placentation, aberrant placental vascularization, maternal hypertensive disorders, and tobacco use, all result in IUGR. Multiple gestation (i.e., twins, triplets) is associated with increased risk of both IUGR and PTB [16]. Infectious diseases, including intrauterine infections, HIV, and malaria, result in LBW due to both growth restriction and short gestation. Multiple maternal characteristics, risk behaviors, and social determinants are associated with both IUGR and PTB; these include maternal short stature, maternal malnutrition, low body mass index, poverty, black race, narrow child spacing, low maternal education, poor antenatal care, substance abuse, and emotional and physical stress [5], [17], [18], [19]. How these factors are mediated biologically remains poorly understood.

Preterm birth may be spontaneous or medically-indicated, such as induction or cesarean section for maternal complications such as pre-eclampsia. Infectious and inflammatory processes are associated with increased risk for PTB, including chorioamnionitis, bacterial vaginosis, bacteriuria, and systemic or remote site infection such as sepsis and periodontal disease.

#### 1.1.3 The importance of short gestation on immune function and vaccine efficacy

Transplacental antibody transfer is an active process mediated by Fc receptors in the placental syncytiotrophoblast [20], which increases from 30 weeks gestation. Small molecular weight particles (<600 Da) cross the placenta by passive mechanism including diffusion, however, larger molecular weight particles (>1000 Da) are transported across the placenta by and active receptor-mediated process [21]. Fetal IgG levels are approximately 50% of maternal antibody level at 32 weeks gestation and rises rapidly through the third trimester [22]. Preterm newborns have significantly lower antibody levels than term newborns [22]. LBW term newborns have significantly lower antibody concentrations to Herpes simplex virus type 1, respiratory syncytial virus ad varicella zoster virus than term newborns with birth weight >2500 g [23].

Maternal antibody levels, receptor density and functionality, avidity, antigen nature, and gestational age determine the efficiency of placental antibody transfer [24]. Diseases that are highly prevalent in some areas, such as malaria and human immunodeficiency virus (HIV), are known to cause placental damage, especially placental malaria [25], [26]. Maternal HIV infection has been consistently associated with reduced placental passage of antibodies against several common viral and bacterial antigens [27], [28]. Placental malaria has been associated with maternal hypergammaglobulinemia and reduced transfer of antibodies against measles virus, *Clostridium tetani*, *Streptococcus pneumonia*, and varicella-zoster virus in some studies [20], [29], [30], [31]. The transfer during pregnancy of maternal antibodies to the fetus minimizes deficiencies in antibody production in the fetus and provides short-term passive immunity [32], conditioning the success of vaccination in newborns [33] which is especially important in preterm and IUGR newborns. Multiple comorbidities are associated with both LBW and immune suppression, such as malnutrition and infection, thereby further exacerbating diminished immune function in the compromised newborn.

#### 1.1.4 Maternal immunization and birth weight

Maternal infections, including influenza, have been associated with increased risk of low birth weight newborns [34]. As a corollary, prevention of certain infections during pregnancy might have a protective effect against LBW. This has been observed in a maternal immunization trial conducted in Bangladesh [35], in which the mean birth weight of infants born to mothers who received an inactivated influenza vaccine during pregnancy was higher than of infants born to mothers who received a pneumococcal polysaccharide vaccine (3178 g vs. 2978 g, p = 0.02). This trend has not been observed in other maternal influenza immunization trials [36].

The field of immunization of pregnant women has highlighted the importance of knowing background rates of adverse pregnancy events, including LBW, PTB, SGA, IUGR, stillbirths, and neonatal death, which can vary markedly between and within regions. The greatest impact of disease prevention from maternal immunization is expected to be observed in LMIC, where the burden of disease is greatest and access to health care services is most limited. For this reason, particular attention is being given to advancing maternal immunization trials in LMICs. Unfortunately, reliable, accurate, and timely reports of vital statistics and demographic data are often limited in these settings.

Data Safety Monitoring Boards are established to review clinical trial data, including regular assessment or review of adverse event rates in trial participants. Without accurate information on background rates of low birthweight and other adverse pregnancy outcomes, it will be impossible to detect an increase in adverse events following immunization. Development of standardized methods to collect and report LBW and other essential outcomes will be essential to advancing maternal immunization programs worldwide.

Birth weight is usually included under demographics of trial participant infants, and the differences in birth weights between participants enrolled in active and placebo or control arms of interventional trials in pregnancy are usually assessed.

The LBW Working Group recommends use of traditional case definitions of LBW as defined by the World Health Organization. This report therefore focuses on delineating data quality related to methods used to estimate birth weight in LMICs, and summarizes some surrogate measurements that are under investigation to assess birth weight and estimate population-level background LBW rates.

### 1.2 Methods for the review of the case definition and guidelines for data collection, analysis, and presentation for low birth weight in clinical trial and population settings

Following the process described in the overview paper [21] as well as on the Brighton Collaboration Website http://www.brightoncollaboration.org/internet/en/index/process.html, the Brighton Collaboration *Low birth weight Working Group* was formed in 2016 and included 16 members of varied backgrounds including clinical, academic, public health and industry. The composition of the working and reference group as well as results of the web-based survey completed by the reference group with subsequent discussions in the working group can be viewed at: http://www.brightoncollaboration.org/internet/en/index/working_groups.html.

To guide the decision-making for the guidelines, a literature search was performed using Medline/PubMed, Embase, ClinicalKey (ebooks), ScienceDirect (eBooks), eBrary (eBooks) and the Cochrane Libraries, including the terms: ‘pregnancy, vaccines and low birth weight’, and restricted to English language publications since 2005. The search resulted in the identification of 41 references. All abstracts were screened for possible reports of Low birth weight following immunization. Thirty-two articles with potentially relevant material were reviewed in more detail, in order to identify studies using case definitions or, in their absence, providing clinical descriptions of the case material. This review resulted in a detailed summary of 19 articles, including information on the study type, the vaccine, the diagnostic criteria or case definition put forth, the time interval since time of immunization, and any other symptoms. Multiple general medical, pediatric and infectious disease book chapters were also searched.

The definition of low birth weight used was consistent across all literature reviewed.

A second literature search using the search terms ‘birth weight and tools’ was performed using Pubmed, to identify other measurements used as proxies for birth weight. The search, unrestricted for language and year of publication, identified in 235 results. Titles were screened and 10 articles were identified for further review.

### 1.3 Rationale for selected decisions about the case definition of low birth weight as an adverse event following maternal immunization

#### 1.3.1 The term low birth weight

‘Low birth weight’ (LBW) has been defined as first weight recorded within hours of birth of <2500 g. Very low birth weight (VLBW) is accepted as <1500 g and extremely low birth weight (ELBW) is <1000 g [1].

Within the definition context, however, the three diagnostic levels must not be misunderstood as reflecting different grades of clinical severity. They instead reflect diagnostic certainty.

The levels of certainty have been formulated such that the Level 1 definition is highly specific for the condition. Two additional diagnostic levels have been included in the definition, offering a stepwise loss of precision and accuracy from Level One down to Level Three, while retaining an approach to expand utilization of available data. In this way it is hoped that information on low birth weight can be captured more broadly at the population level.

#### 1.3.2 Timing of birth weight assessment

The birth weight is described as the first weight measured, however, in settings with low rates of facility-based deliveries, a newborn may not be assessed by a health care worker until several days old. Birth weight should be assessed within hours of birth, prior to significant weight loss [37]. Term neonates lose between 3.5% and 6.6% of their birth weight within the first 2.5–2.7 days of life. Exclusively breastfed neonates have a greater weight loss (Median 6.6%, 95%CI 6.3–6.9%) than formula-fed (Median 3.5%, 95%CI 3.0–3.9%) or mixed fed (5.9%, 95%CI 4.8–6.9%) neonates respectively, and take longer to regain their birth weight (8.3 vs. 6.5 vs. 7.9 days) [37].

The LBW working group decided to restrict ‘birth weight’ to a weight measured in the first 48 h of life. In the absence of a weight measured within the first 48 h of life, a weight measured during the first week of life, could be classified as an ‘early neonatal weight’ but not ‘birth weight’.

In a clinical trial scenario, measurement of weight within first 48 h of life should be achievable, as the clinical trial would procure adequate equipment, employ and train staff to assess birth weight in a timely manner, and enroll participants who reside in areas which are relatively easily accessed by trial or health care staff.

Many newborns globally are not weighed within hours of birth, mainly due to difficulty in accessing health care personnel, facilities, and essential equipment. Specific time frames for onset of symptoms following immunization are not included for the following main reasons:

We postulate that a definition designed to be a suitable tool for testing causal relationships requires ascertainment of the outcome (e.g. low birth weight) independent from the exposure (e.g. immunizations). Therefore, to avoid selection bias, a restrictive time interval from immunization to birth of a LBW newborn should not be an integral part of such a definition. Instead, where feasible, details of this interval should be assessed and reported as described in the data collection guidelines.

Further, measurement of birth weight often occurs outside the controlled setting of a clinical trial or hospital. In some settings it may be impossible to obtain a clear timeline of the assessment of a birth weight, particularly in less developed or rural settings. In order to avoid selecting against such cases, the Brighton Collaboration case definition avoids setting arbitrary time frames. The time between delivery and measurement of birth weight should be recorded and accounted for in the analysis.

### 1.4 Guidelines for data collection, analysis and presentation

As mentioned in the overview paper [38], the case definition is accompanied by guidelines which are structured according to the steps of conducting a clinical trial, i.e. data collection, analysis and presentation. Neither case definition nor guidelines are intended to guide or establish criteria for management of ill infants, children, or adults. Both were developed to improve standardization of case definitions and data comparability.

### 1.5 Periodic review

Similar to all Brighton Collaboration case definitions and guidelines, review of the definition with its guidelines is planned on a regular basis (i.e. every three to five years) or more often if needed.

## 2 Case definition of low birth weight^3^

**Level 1 of diagnostic certainty**

**Table:** 

Newborn infant weighed within 24 h of birth	AND
Use electronic scale which is graduated to 10 g	AND
Scale is calibrated at least once a year	AND
Scale placed on level, hard surface	AND
Scale tared to zero grams	AND
Weight recorded as <2500 g	OR
Birth weight recorded as <2500 g	AND
Birth weight assessed as per health care facility’s standard operating procedure, which fulfills criteria 1 to 5 of LOC1	

**Level 2 of diagnostic certainty**

**Table:** 

Newborn infant weighed within 24 h of birth	AND
Scale (electronic/spring) is graduated to at least 50 g	AND
Scale is calibrated at least once a year, or more often if moved	AND
Scale tared to zero grams or 0.00 kg	AND
Weight recorded as <2500 g	OR
Birth weight recorded as <2500 g	AND
Birth weight assessed as per health care facility’s standard operating procedure, which fulfills criteria 1 to 4 of LOC2	

Scale used: could be electronic or spring scale, including color-coded scale.

**Level 3 of diagnostic certainty**

**Table:** 

Newborn infant weighed on day 1 or 2 of life (first 48 h of life)	AND
Weight measured using dial/spring/color-coded scale	AND
Weight assessed as <2500 g	

**Level 4 of diagnostic certainty**

**Table:** 

Newborn infant ‘weight’ assessed on day 1 or 2 of life (first 48 h of life)	AND
Proxy measure of birth weight used	AND
Weight CATEGORY assessed as <2500 g	

In many settings, including high-income countries, birth weight is assessed by a health care provider who is attendant during/soon after delivery, and not the vaccine trialist/researcher. The details of time of birth weight assessment, and details of scale used and calibration details are usually not recorded in newborn assessment medical notes.

The newborn weight assessment is presumed to be assessed accurately as per health care center’s standard operating procedures. In many instances, trialists need to rely on the attending medical staff at health care facility for birth weight assessment. Strengthening training and oversight of birth weight measurement would be expected to strengthen data both in clinical trials and post-marketing surveillance.

### 2.1 Other tools under investigation to estimate birth weight in individuals and populations

Up to 60 million infants are born at home annually [39], and up to 48% of infants worldwide are not weighed at birth [3]. Lack of access to health care facilities or health care workers hampers accurate assessment of low birth weight rates in many regions. In order to identify small newborns, who could be preterm, IUGR, or both, who require additional care, inexpensive tools are required which can be utilized in the field.

The lack of data available has encouraged the development of a mathematical model to calculate the expected number of adverse events, including neonatal and maternal deaths, SGA, preterm birth and major congenital malformations [40].

Several anthropometric measurements, including chest circumference, foot length and mid-upper arm circumference, have been assessed as proxies for birth weight [41], [42], [43], [44]. Table 1 summarizes these tools and their validity for identifying low birth weight newborns. These tools at this point are considered investigational and have been included in level 4 definition only, which indicates that evidence is inadequate to meet the definition, however, may be useful for population background LBW estimates.

**Table 1:** Validated tools used as proxy measures of birth weight.

	Measurement	Method of assessment	Cut-off values used	Comments
Newborn foot length [41], [42], [43], [46]	Foot length from center of heel pad to tip of big toe in millimeters	Hard plastic ruler pressed vertically against sole of foot (highest AUC)	7.2 cm for 2000 g	Weakest correlation with LBW of all anthropometric measurements [47], [48]
		Sole of foot placed on solid board with measuring tape	7.8 cm for preterm [41]	
			⩽7.4 cm (7.3–7.4 cm) for 2500 g [43]	AUC 0.94, 95%CI 0.92–0.96 [43]
			For <2500 g	
		Footprint made on White paper, and tip of big toe and heel marked with pencil	7.2 cm (Europe)	<8 cm at birth was 87% sensitive for LBW [46]
			6.3–7.85 cm (Asia)	
			7.4–8 cm (Africa)	
Chest circumference [42], [43]	Chest circumference at level of nipples in centimeters	Non-elastic, flexible measuring tape graduated to nearest 0.1 cm, measured during expiration	⩽30.4 cm (30.0–30.4 cm) [43]	Highly predictive of LBW if measured at <24 h of age (AUC 0.98, 95%CI 0.96–0.99) [43]
				In meta-analysis, best anthropometric measurement to predict LBW [47]
				Risk of hypothermia
Mid upper arm circumference [43]	Mid-point between tip of acromion process and olecranon process in centimeters	Non-elastic, flexible measuring tape graduated to nearest 0.1 cm	⩽9.0 cm (8.7–9.0 cm) [43]	Highly predictive of LBW if measured at <24 h of age (AUC 0.98, 95%CI 0.96–0.99) [43]

AUC – area under curve.

**Fig. 1:**
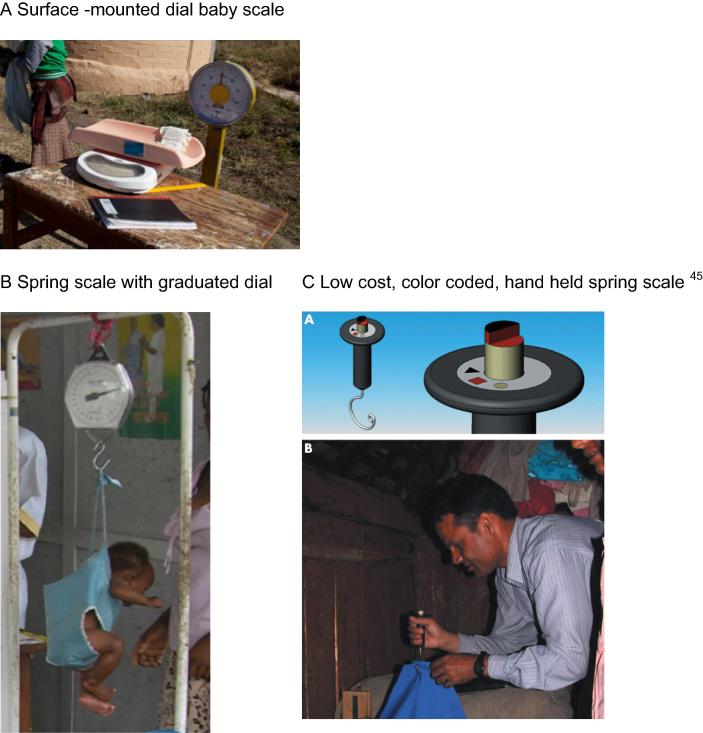
Tools used to measure birth weight (See above-mentioned references for further information.).

In addition to these measurements, other tools are utilized in some communities to assess birth weight, including difference between adult weight with and without newborn in arms (see Fig. 1).

## 3 Guidelines for data collection, analysis and presentation of low birth weight

It was the consensus of the Brighton Collaboration *Working Group* for Low birth weight to recommend the following guidelines to enable meaningful and standardized collection, analysis, and presentation of information about low birth weight. However, implementation of all guidelines might not be possible in all settings. The availability and quality of information may vary depending upon resources, geographical region, and whether the source of information is a prospective clinical trial, epidemiological study, post-marketing surveillance, or an individual report. Also, as explained in more detail in the overview paper [38], these guidelines have been developed by this working group for guidance only, and are not to be considered a mandatory requirement for data collection, analysis, or presentation.

### 3.1 Data collection

These guidelines represent a desirable standard for the collection of data on availability following immunization to allow for comparability of data, and are recommended as an addition to data collected for the specific study question and setting. The guidelines are not intended to guide the primary reporting of low birth weight to a surveillance system or study monitor. Investigators developing a data collection tool based on these data collection guidelines also need to refer to the criteria in the case definition, which are not repeated in these guidelines.

Guidelines numbers below have been developed to address data elements for the collection of adverse event information as specified in general drug safety guidelines by the International Conference on Harmonization of Technical Requirements for Registration of Pharmaceuticals for Human Use [49], and the form for reporting of drug adverse events by the Council for International Organizations of Medical Sciences [50]. These data elements include an identifiable reporter and patient, one or more prior immunizations, and a detailed description of the adverse event, in this case, of low birth weight following immunization. The additional guidelines have been developed as guidance for the collection of additional information to allow for a more comprehensive understanding of low birth weight following maternal immunization.

#### 3.1.1 Source of information/reporter

For all cases and/or all study participants, as appropriate, the following information should be recorded:

(1) Date of report.
(2) Name and contact information of person reporting^4^ and/or diagnosing low birth weight as specified by country-specific data protection law.
(3) Name and contact information of the investigator responsible for the subject, as applicable.
(4) Relation to the patient (e.g., healthcare provider, immunizer, community health worker, family member [indicate relationship], other).

#### 3.1.2 Vaccinee/control

##### 3.1.2.1 Demographics

For all cases and/or all study participants, as appropriate, the following information should be recorded:

(5) Case/study participant identifiers for mother and newborn (e.g. first name initial followed by last name initial) or code (i.e. hospital identifier or in accordance with country-specific data protection laws). Each newborn should have a unique identifier, ideally linked to mother’s identifier (e.g. participant code could be same for mother and baby(ies), with an added prefix/suffix to identify mother/baby).
(6) Maternal date of birth, or if not available, maternal age.
(7) For each infant: Date and time of delivery, single or multiple, live birth vs. fetal death (fresh or macerated), estimated gestational age, method of determination of gestational age (LMP, fundal height, first trimester ultrasound) and birth weight.
  • For collection of birth weight, ideally record timeline of weight measurement (e.g. time of delivery to time of weight), type of scale used (e.g. surface-mounted spring) and place where birth weight was measured (e.g. health care facility, mobile health worker visiting home).

##### 3.1.2.2 Clinical and immunization history

For all cases and/or all study participants, as appropriate, the following information should be recorded:

(8) Maternal past medical history, including hospitalizations, gravidity and parity, underlying diseases/disorders; complications of pregnancy, labor, or delivery; pre-immunization signs and symptoms including identification of indicators for, or the absence of, a history of allergy to vaccines, vaccine components or medications; food allergy; allergic rhinitis; eczema; asthma.
(9) Any medication history (other than treatment for the event described) prior to, during, and after immunization including prescription and non-prescription medication as well as medication or treatment with long half-life or long term effect. (E.g. immunoglobulins, blood transfusion and immunosuppressants).
(10) Immunization history (i.e. previous immunizations and any adverse event following immunization (AEFI)), in particular occurrence of low birth weight after a previous maternal immunization.

#### 3.1.3 Details of the immunization

For all cases and/or all study participants, as appropriate, the following information should be recorded:

(11) Date and time of maternal immunization(s).
(12) Description of vaccine(s) (name of vaccine, manufacturer, lot number, dose (e.g. 0.25 mL, 0.5 mL), vaccine diluent (composition and lot number) and number of dose if part of a series of immunizations against the same disease).
(13) The anatomical sites (including left or right side) of all immunizations (e.g. vaccine A in proximal left lateral thigh, vaccine B in left deltoid).
(14) Route and method of administration (e.g. intramuscular, intradermal, subcutaneous, and needle-free (including type and size), other injection devices).
(15) Needle length and gauge.

#### 3.1.4 The adverse event

(16) For all cases at any level of diagnostic certainty and for reported events with insufficient evidence, the criteria fulfilled to meet the case definition should be recorded.

Specifically document:

(17) Severity of Low birth weight (LBW, VLBW or ELBW), and if there was medical confirmation of the LBW (i.e. patient seen by physician/other health care worker).
(18) Date/time of observation,^5^ and diagnosis.^6^
(19) Concurrent signs, symptoms, and diseases, including prematurity.
(20) Measurement/testing.
  • Values and units of routinely measured parameters (grams for birth weight);
  • Method of measurement (e.g. type of scale.);
  • Weight should be recorded with minimal or ideally no clothing;
(21) Objective clinical evidence supporting classification of the event as “serious”.^7^
(22) Exposures other than the immunization 24 h before and after immunization (e.g. infection, environmental) considered potentially relevant to the reported event.^8^

#### 3.1.5 Miscellaneous/general

(23) The duration of surveillance for low birth weight should be from 0 to 48 h of life. Any weight measured after 48 h of age should not be considered a ‘birth weight’.^9^
(24) Methods of data collection should be consistent within and between study groups, if applicable.^10^
(25) Investigators of patients with low birth weight should provide guidance to reporters to optimize the quality and completeness of information provided.

### 3.2 Data analysis

The following guidelines represent a desirable standard for analysis of data on low birth weight to allow for comparability of data, and are recommended as an addition to data analyzed for the specific study question and setting.

(26) Reported events should be classified in one of the following five categories including the three levels of diagnostic certainty. Events that meet the case definition should be classified according to the levels of diagnostic certainty as specified in the case definition. Events that do not meet the case definition should be classified in the additional categories for analysis.

**Event classification in 5 categories**

**Event meets case definition**

(1) Level 1: Criteria as specified in the Low birth weight case definition
(2) Level 2: Criteria as specified in the Low birth weight case definition
(3) Level 3: Criteria as specified in the Low birth weight case definition

**Event does not meet case definition**

***Additional categories for analysis***

(4) Reported Low birth weight with insufficient evidence to meet the case definition.^7^
(5) Birth weight not assessed, therefore data unavailable.
(27) The interval between immunization and reported Low birth weight could be defined as the date/time of immunization to the date/time of assessment^4^ of birth weight. If few cases are reported, the concrete time course could be analyzed for each; for a large number of cases, data can be analyzed in the following increments.
(28) If birth weight is assessed by more than one method, the value recorded which fulfills the highest level of certainty should be used as the basis for analysis.
(29) The distribution of birth weight data could be analyzed in predefined increments (e.g. LBW < 2500 g, VLBW < 1500 g, ELBW < 1000 g). Increments specified above should be used. When only a small number of cases are presented, the respective values can be presented individually.
(30) Data on Low birth weight obtained from participants whose mothers received a vaccine should be compared with those obtained from an appropriately selected and documented control group to assess background rates of LBW in non-exposed populations, and should be analyzed by study arm and dose where possible, e.g. in prospective clinical trials.

### 3.3 Data presentation

These guidelines represent a desirable standard for the presentation and publication of data on Low birth weight following immunization to allow for comparability of data, and are recommended as an addition to data presented for the specific study question and setting. Additionally, it is recommended to refer to existing general guidelines for the presentation and publication of randomized controlled trials, systematic reviews, and meta-analyses of observational studies in epidemiology (e.g. statements of Consolidated Standards of Reporting Trials (CONSORT) [51], of Improving the quality of reports of meta-analyses of randomized controlled trials (QUORUM) [52], and of meta-analysis Of Observational Studies in Epidemiology (MOOSE) [53], respectively).

(31) All reported events of Low birth weight should be presented according to the categories listed in guideline 31.
(32) Data on Low birth weight events should be presented in accordance with data collection guidelines 1–25 and data analysis guidelines 26–30.
(33) Data should be presented as rates with a numerator and denominator (n/N) (and not only in percentages), with confidence intervals around the point estimates.

Although immunization safety surveillance systems denominator data are usually not readily available, attempts should be made to identify approximate denominators. The source of the denominator data should be reported and calculations of estimates be described (e.g. manufacturer data like total doses distributed, reporting through Ministry of Health, coverage/population based data, etc.).

(34) The incidence of cases in the study population should be presented and clearly identified as such in the text.
(35) If the distribution of birth weight data is skewed, median and range are usually the more appropriate statistical descriptors than a mean. However, the mean and standard deviation should also be provided.
(36) Any publication of data on Low birth weight should include a detailed description of the methods used for data collection and analysis as possible. It is essential to specify:
  • The study design;
  • The method, frequency and duration of monitoring for Low birth weight;
  • The trial profile, indicating participant flow during a study including drop-outs and withdrawals to indicate the size and nature of the respective groups under investigation;
  • The type of surveillance (e.g. passive or active surveillance);
  • The characteristics of the surveillance system (e.g. population served, mode of report solicitation);
  • The search strategy in surveillance databases;
  • Comparison group(s), if used for analysis;
  • The instrument of data collection (e.g. standardized questionnaire, diary card, report form);
  • Whether the day of immunization was considered “day one” or “day zero” in the analysis;
  • Whether the date of onset^4^ and/or the date of first observation^5^ and/or the date of diagnosis^6^ was used for analysis; and
  • Use of this case definition for Low birth weight, in the abstract or methods section of a publication.^11^

## Disclaimer

The findings, opinions and assertions contained in this consensus document are those of the individual scientific professional members of the working group. They do not necessarily represent the official positions of each participant’s organization (e.g., government, university, or corporation). Specifically, the findings and conclusions in this paper are those of the authors and do not necessarily represent the views of their respective institutions.

## Footnotes

3 The case definition should be applied when there is no clear alternative diagnosis for the reported event to account for the combination of symptoms.

4 If the reporting center is different from the vaccinating center, appropriate and timely communication of the adverse event should occur.

5 The date and/or time of observation is defined as the time post immunization, when the Low birth weight was recorded.

6 The date of diagnosis of an episode is the day post immunization when the event met the case definition at any level.

7 An AEFI is defined as serious by international standards if it meets one or more of the following criteria: (1) it results in death, (2) is life-threatening, (3) it requires inpatient hospitalization or results in prolongation of existing hospitalization, (4) results in persistent or significant disability/incapacity, (5) is a congenital anomaly/birth defect, (6) is a medically important event or reaction.

8 To determine the appropriate category, the user should first establish, whether a reported event meets the criteria for the lowest applicable level of diagnostic certainty, e.g. Level three. If the lowest applicable level of diagnostic certainty of the definition is met, and there is evidence that the criteria of the next higher level of diagnostic certainty are met, the event should be classified in the next category. This approach should be continued until the highest level of diagnostic certainty for a given event could be determined. Major criteria can be used to satisfy the requirement of minor criteria. If the lowest level of the case definition is not met, it should be ruled out that any of the higher levels of diagnostic certainty are met and the event should be classified in additional categories four or five.

9 If the evidence available for an event is insufficient because information is missing, such an event should be categorized as “Reported Low birth weight with insufficient evidence to meet the case definition”.

10 An event does not meet the case definition if investigation reveals a negative finding of a necessary criterion (necessary condition) for diagnosis. Such an event should be rejected and classified as “Not a case of Low birth weight”.

11 Use of this document should preferably be referenced by referring to the respective link on the Brighton Collaboration website (http://www.brightoncollaboration.org).
